# An Improved Model of Physical and Emotional Social Defeat: Different Effects on Social Behavior and Body Weight of Adolescent Mice by Interaction With Social Support

**DOI:** 10.3389/fpsyt.2018.00688

**Published:** 2018-12-11

**Authors:** Man Li, Hang Xu, Weiwen Wang

**Affiliations:** ^1^Department of Psychology, Tianjin Normal University, Tianjin, China; ^2^Academy of Psychology and Behavior, Tianjin Normal University, Tianjin, China; ^3^Center of Collaborative Innovation for Assessment and Promotion of Mental Health, Tianjin, China; ^4^CAS Key Laboratory of Mental Health, Institute of Psychology, Beijing, China; ^5^Department of Psychology, University of Chinese Academy of Sciences, Beijing, China

**Keywords:** adolescent, physical stress, emotional stress, social behavior, social support

## Abstract

Social stress is a prevalent etiological environmental factor that can affect health, especially during adolescence. Either experiencing or witnessing a traumatic event during adolescence can increase the risk of psychiatric disorders, such as PTSD. The present study attempted to establish an improved social stress model to better distinguish the effects of physical and emotional social stress on the behavior and physiology of adolescent mice. In addition, we investigated how social support affected these stress-induced changes in social behavior. On PND 28, male littermates were exposed to either physical stress (PS) or emotional stress (ES), afterwards, half of them were paired-housed and the others were singly housed. The PS exposed mice were directly confronted with a violent aggressor using the social defeat stress (SDS) paradigm for 15 min/trial (with the total of 10 trials randomly administered over a week), while the ES exposed mice were placed in a neighboring compartment to witness the PS procedure. Our results indicate that both stressors induced an effective stress response in adolescent mice, but PS and ES had differential influence in the context of relevant social anxiety/fear and social interaction with peers. Additionally, social support following stress exposure exerted beneficial effects on the social anxiety/fear in ES exposed mice, but not on PS exposed mice, suggesting that the type of stressor may affect the intervention efficacy of social support. These findings provide extensive evidence that physical and emotional stressors induce different effects. Moreover, ES exposed mice, rather than PS exposed mice, seemed to benefit from social support. In summary, the study suggests that this paradigm will be helpful in investigating the effects of psychological intervention for the treatment of stress-related psychiatric disorders.

## Introduction

Adolescence, a transition period between childhood and adulthood, is a critical period for the development of social psychology and the brain (1, 2). During this period, an individuals' social relations experience a transition from family-oriented to peer-, school-, and environment-directed relations, showing strong interest in and sensitivity to social information, such as social interaction, novelty seeking, etc. (3). Concomitant with social behavioral manifestations in adolescence, the structure and function of the social brain, which refers to the network of brain regions underlying social emotion and cognition, especially the prefrontal cortex and subcortical pathways, undergo profound and rapid developmental changes (4). These constitute the unique neurobehavioral characteristics of adolescents, that also increase their vulnerability to a variety of social stressors (5, 6). For instance, it has been indicated that a variety of negative social experiences, including peer bullying, abuse, etc., can act as substantial stressors in the adolescent group (7, 8) and intimately relate to the onset of psychiatric disorders, such as PTSD, depression, etc. (9–11).

It is not only experiencing traumatic events, but also witnessing such events that can increase the risk of psychiatric disorders, such as PTSD. Previous studies showed that approximately 25–30% of individuals who witnessed a traumatic event might develop PTSD and other forms of mental disorders, including depression (12, 13). In animal studies, it has also been observed that exposing mice to both physical stress (e.g., foot shocks) and emotional stress (e.g., witnessing foot shocks) induced conditional fear memory, a core symptom of PTSD (14, 15). Although the foot-shock model is useful for investigating the effects of PS and ES, it is a rather severe stressor and is difficult to translate to the human context. Peer bullying, on the other hand, is a common stressor in children and adolescents, shown to be highly predictive of subsequent psychopathology, such as PTSD (16–18). Such types of socially stressful experiences in humans can be simulated by the social defeat stress (SDS) model in rodents, typically by the “resident-intruder” paradigm. It has been proven that this model can effectively induce emotional and cognitive alterations relevant to symptoms of a patient with stress-related psychiatric disorders, such as PTSD and depression (19). The protocol of SDS often includes two stages: first, the experimental subjects (the “intruders”) are directly exposed to the aggressive subjects (the “resident”) to induce PS for a short time (usually, 5–15 min); afterwards, they are separated by a transparent, perforated divider to maintain sensory contact for a period of time (e.g., the remainder of the day, or 30 min, etc.) (13, 19, 20;), thereby inducing further emotional stress in the defeated animal. However, the model can be adapted to include a pure ES group composed of animals that witness the social defeat of others. Warrant et al. observed that mice in both PS and ES groups showed a smaller weight gain, decreased social interaction and increased anxiety-like behaviors in the elevated-plus maze (EPM) test after 7 days of stress exposure (20). Miao et al. also found that pregnant mice exhibited decreased sucrose preference and spent less time in the open arms of the EPM after witnessing the defeat of their mates (21). However, as mentioned above, the PS animals are in fact exposed to both PS (in the first stage of SDS), and ES (in the second stage of SDS). Therefore, it is difficult to distinguish between the separate effects of PS and ES on behavior and physiology. Due to a common etiological stressor, it is necessary to develop an improved PS and ES model to further clarify the effects of the two types of stressors.

There is extensive evidence that social support is an important factor affecting the consequences of stress. Substantive social support is known to be a protective factor decreasing or preventing the detrimental effects of stress, especially under conditions of severe stress (22). In animal research, isolation-housing after SDS induces prolonged behavioral and physiological alterations, including reduced sucrose preference, cognitive impairment, enhanced anxiety-like behavior in the EPM test, social avoidance, enhanced locomotor activity in the open field test and an increased heart rate, etc.(23–25). Intriguingly, these effects were substantially reduced in animals that were group-housed after SDS. These studies clearly indicate that social relations and/or social support can play an important role in reducing the effect of stress. However, not all studies have reached this conclusion and some have even indicated that social relationships might act as a new stressor, under certain conditions (26, 27). Moreover, it remains to be explored whether social support has a similar effect in PS- and ES-exposed animals.

As social stress is an important etiological factor in the (mental) health of adolescents, the present study aimed to establish an improved model on the basis of classical social defeat stress, to better distinguish the effects of PS and ES on social behavior. We also investigated the role of social support in these effects.

## Materials and Methods

### Animals

Male offspring of C57BL/6J mice (Beijing Vital River Laboratory Animal Technology Co., Ltd) obtained at weaning (postnatal day, PND21) from our in-house breeding program (Center for Experimental Animal Research, Institute of Psychology, Chinese Academy of Sciences) were used as intruders. From PND21 to PND28, male siblings were housed in groups of 2-4 mice per cage, with free access to water and food. Male CD-1 mice (Beijing Vital River Laboratory Animal Technology Co., Ltd) were used as residents and housed singly until 3–4 months of age. All mice were bred and housed under standard conditions [12:12 h light-dark cycle with lights on at 07:00 a.m.; the room temperature was 20 ± 2°C].

Experimental procedures were performed with the approval of the Institutional Review Board of the Institute of Psychology, Chinese Academy of Health Guide for the Care and Use of Laboratory Animals.

### Physical and Emotional Stress Procedures

**Figure 1:**
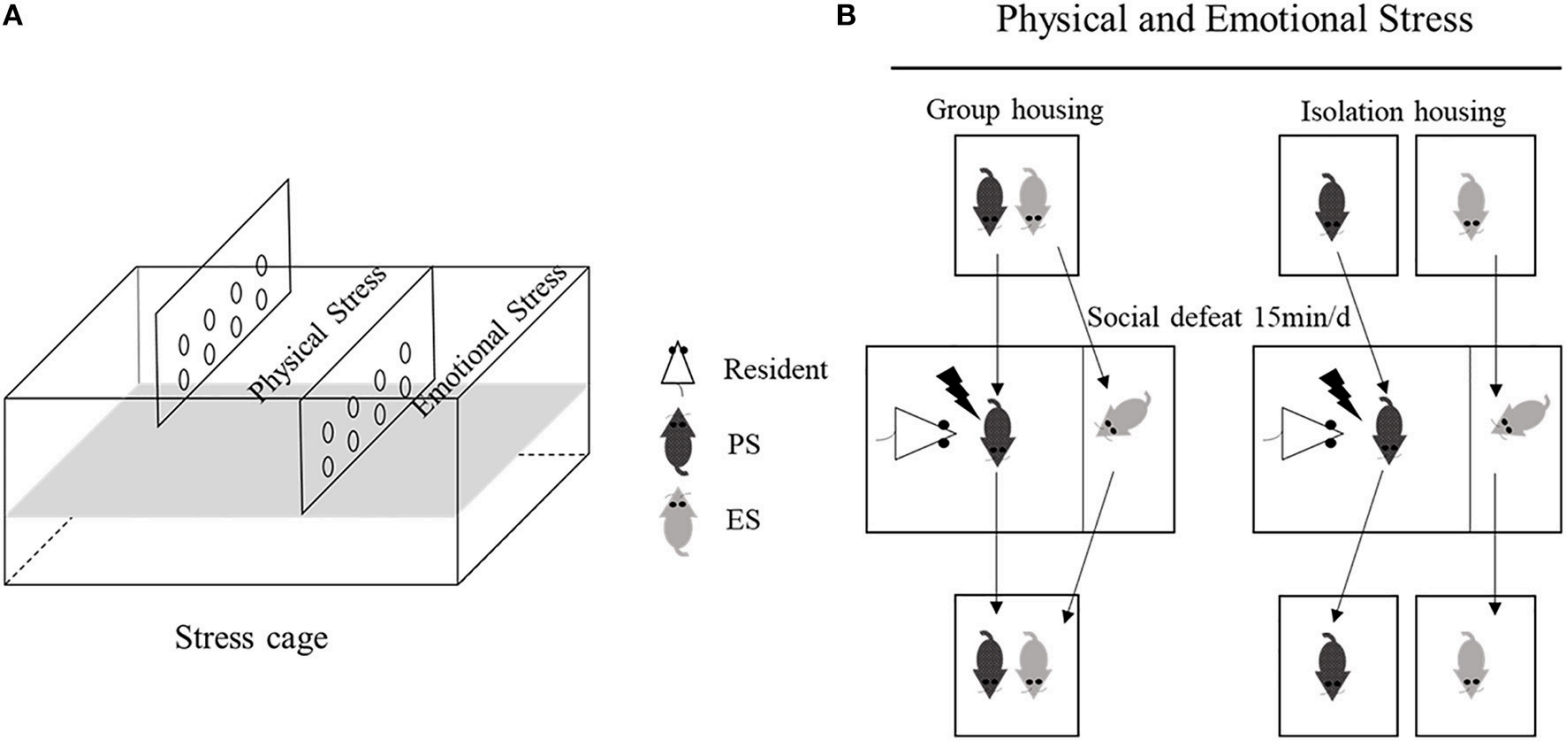
Schematic representation of the stress cage **(A)**, and the physical and emotional stress process **(B)**.

The stress cage (L × W × H: 45 × 30 × 17.5 cm) was divided into three equal chambers (L × W × H: 45 × 10 × 11 cm) with transparent perforated Plexiglas boards (Figure 1A). Appropriate selection of aggressive CD-1 male mice is critical for the successful application of physical and emotional stress. Therefore, CD-1 mice were selected as aggressors (residents) based on the following standards: attack latency shorter than 10 s and multiple or continuous violent attacks for three consecutive days. Additionally, prior to the stress test, the resident CD-1 mice were placed in the stress cage for at least 3 days to enhance their territorial behavior.

On PND28, male litters were assigned to physical stress (PS, *n* = 14) or emotional stress (ES, *n* = 14) groups. As the social relationship (e.g., familiar or unfamiliar) is an important factor of modulating the intensity of emotional stress (28, 29), littermates were paired into PS and ES groups. PS mice were directly placed in the stress cage, where they experienced physical aggression by a CD-1 resident for 15 min, similar to the “resident-intruder” paradigm described previously (30, 31); meanwhile, the ES littermates witnessed the social defeat process in the adjacent chamber (Figure 1B). After each social defeat exposure, the PS and ES mice were returned to their home cages; half of them were housed in pairs, and the others were singly housed. PS mice faced different residents each time and a total of 10 randomized social defeats over 1 week were performed, to maintain the stress effect (32). The stress submission was performed in the morning or the afternoon of a given day, according to a randomized defeat time and frequency and two defeats were administered on three randomly selected days over 1 week, with one defeat on the remaining 4 days. During the entire stress period, mice in the control (CON) group were placed in the same cage while the residents were C57BL/6J mice and were separated by the dividers in the stress cages, to avoid direct physical contact. No aggressive behavior occurred during these control sessions.

Body weight was recorded before the social defeat and after the last social defeat protocol. The behavioral tests were conducted 24 h after the last social defeat stress.

### Behavioral Tests

#### Three-Chamber Social Approach Test

A modification of the sociability and social novelty preference test was used to reflect the level of social interest and the ability to recognize new social objects (33). The testing apparatus was a three-chamber rectangular arena (L × W × H: 60 × 40 × 20 cm, made of white Plexiglas), that was divided into three equal zones by two transparent Plexiglas partitions. There was a channel that could be closed and opened (8 × 8 cm) at the bottom of the partition to allow the mouse to move between chambers (Supplementary Figure 1). The distance traveled and the time spent in each zone, were automatically recorded by infrared video tracking and analysis in the dark condition (EthoVision XT with Social Interaction Module; Noldus Information Technology).

The test consisted of three stages. After each stage, the mouse was returned to the home cage. There was a 5 min interval between each stage.

In the first stage, the shuttle channel was closed. The mouse was placed in the middle chamber and allowed to explore for 10 min. The distance traveled in the middle zone was recorded to assess the locomotor activity. The second stage was the social interaction test. Two unfamiliar C57BL/6J males that had no prior contact with the subject mice were placed in the wire cage (L × W × H: 20 × 10 × 10 cm) in the corner of each interaction zone. The shuttle channels were opened, and the experimental mouse was placed in the same starting position, in the middle zone and allowed to explore freely for 10 min. The third stage was the new social object recognition test. To avoid the influence of position preference, a new strange mice was placed in the wire cage on one side where the experimental mouse spent the least amount of time in the second stage. Afterwards, the mouse was placed in the middle zone at the same starting position and allowed to explore freely for 10 min. The chambers of the testing apparatus were cleaned with 75% ethanol to prevent olfactory interference with subsequent tests.

The time spent in each zone was recorded. The social interaction (SI) was calculated as (time spent in the two interaction zones)/(time in the middle zone). The new social object recognition was calculated as (time spent in the new object zone)/(total time spent in the interaction zone), which also reflects the level of social working memory.

#### Social Avoidance

The next day after the three-chamber social approach tests, social avoidance was tested as described previously (19, 30). In brief, mice were placed in an arena (L × W × H: 40 × 40 × 20 cm) that contained an interaction zone (L × W: 20 × 14 cm) at one end of the arena with a small, metallic mesh cage (L × W × H: 8 × 8 × 8 cm) in the middle. The time spent in the interaction zone was initially monitored for 150 s in the absence of a CD-1 mouse, followed by another 150 s in the presence of a CD-1 mouse in the small cage, which was automatically recorded by an infrared behavior tracking and analysis system (EthoVision XT with Social Interaction Module; Noldus Information Technology). The social avoidance ratio was calculated as (time in the interaction zone with CD-1)/(time in the interaction zone without CD-1). After each test, the arena was cleaned with 75% ethanol to prevent olfactory interference with subsequent tests.

### Statistical Analysis

Results are expressed as the mean ± SEM. The analysis was performed using the GraphPad Prism6 software (USA). The weight data and the behavioral data for locomotor activity, social interaction ratio, working memory and social avoidance were analyzed using a two-way ANOVA. Following significant results from the analysis of variance, an LSD analysis was used as the *post hoc* test. The significance level was defined as a *p* < 0.05.

## Results

### Effects of Stress and Rearing Condition on Body Weight Gain

**Figure 2:**
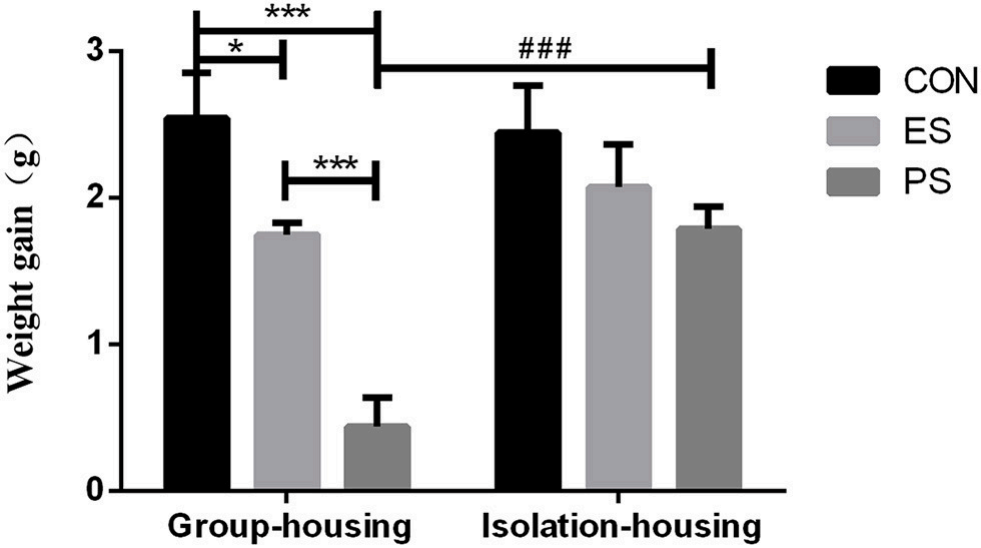
Effects of stress and housing conditions on the bodyweight gain. The results are expressed as the mean ± S.E.M (*n* = 7 per group). ***p < 0.001, **p < 0.01, * *p* < 0.05 indicate the *p*-value for the differences among CON, PS, and ES mice. ^###^*p* < 0.001 corresponds to the difference between housing conditions in the PS group.

As shown in Figure 2, the two-way ANOVA indicated significant main effects of stress (*F*_(2, 37)_ = 16.45, *p* < 0.001) and housing conditions (*F*_(1, 37)_ = 7.68, *p* < 0.05) as well as stress × housing condition interaction (*F*_(2, 37)_ = 4.67, *p* < 0.05). The *post hoc* analysis showed that both PS and ES significantly reduced the bodyweight gain under the group housing condition compared to the controls (*p* < 0.05) and the PS mice experienced the least weight gain. However, isolation housing increased the body-weight gain significantly in PS mice compared to the group housing condition (*p* < 0.001).

### Effects of Stress and Housing Conditions on Locomotor Activity

**Figure 3:**
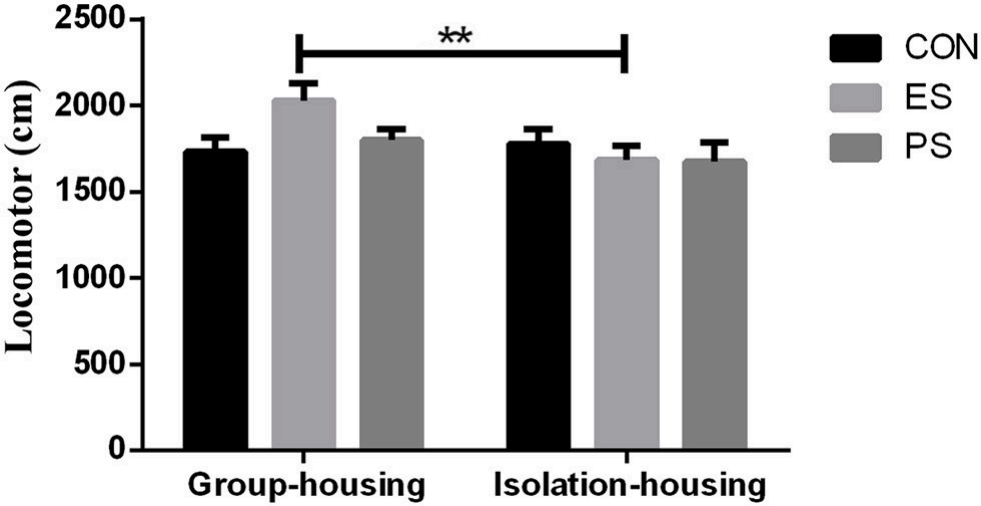
Effects of stress and housing conditions on locomotor activity. The results are expressed as the mean ± S.E.M (*n* = 7 per group), with **p < 0.01 for the difference between housing conditions in the ES group.

As shown in Figure 3, there were marginally significant main effects of housing conditions (*F*_(1, 37)_ = 3.86, *p* = 0.058), but not of stress (*F*_(2, 37)_ = 1.08, *p* = 0.35) or of stress × housing condition interaction (*F*_(2, 37)_ = 2.56, *p* = 0.091). The *post hoc* analysis indicated that mice in the group housing condition in the ES group exhibited significantly increased locomotor activity compared to those in the ES isolation housing condition (*p* < 0.01).

### Effects of Stress and Housing Conditions on Social Interaction

**Figure 4:**
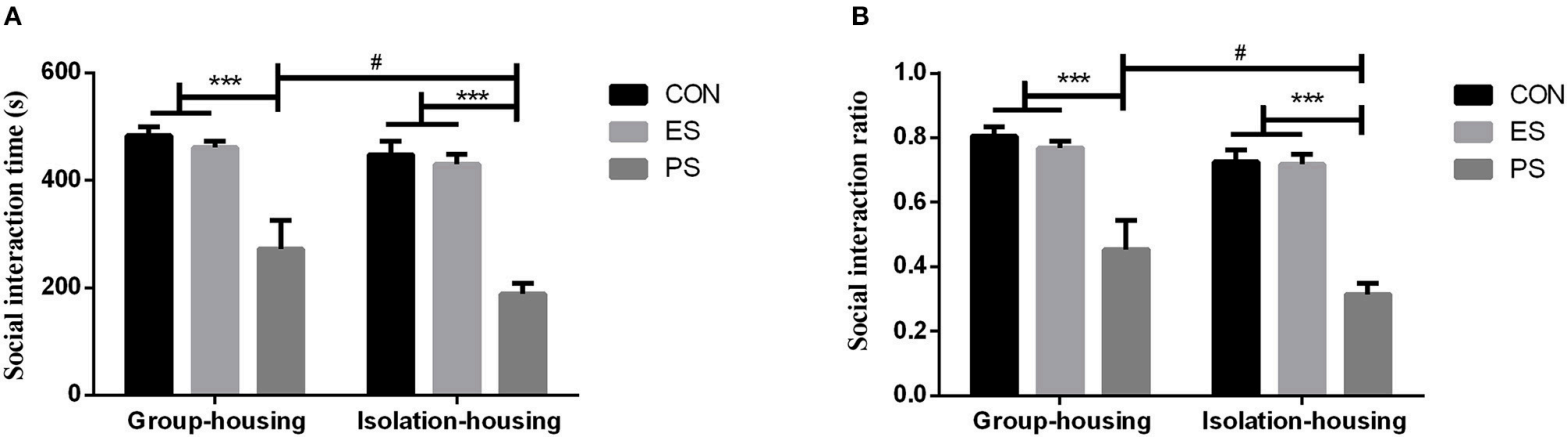
Effects of stress and housing conditions on social interaction time **(A)** and ratio **(B)**. The results are expressed as the mean ± S.E.M (*n* = 7 per group). The social interaction time is calculated as a sum of the time spent in the two interaction zones. The social interaction ratio was defined as (time spent in the two interaction zones)/(time in the middle zone) × 100%. ***p < 0.001, compared to controls; ^#^*p* < 0.05 for the difference between housing conditions in the PS group.

The social interaction performance is shown in Figure 4. There were significant main effects of stress (time: *F*_(2, 37)_ = 40.78, *p* < 0.001; ratio: *F*_(2, 37)_ = 43.64, *p* < 0.001) and housing conditions (time: *F*_(1, 37)_ = 4.35, *p* < 0.05; ratio: *F*_(1, 37)_ = 5.58, *p* < 0.05) but not of stress × housing conditions interaction (time: *F*_(2, 37)_ = 0.51, *p* = 0.60; ratio: *F*_(2, 37)_ = 0.46, *p* = 0.63). The *post hoc* analysis revealed that only mice that experienced physical stress showed a significant reduction in social interaction in both the group and isolation housing conditions (*p* < 0.05). Although the interaction between the housing condition and stress was not significant, the mice housed in isolation had a more pronounced reduction of social interaction in the PS group (*p* < 0.05).

### Effects of Stress and Housing Conditions on New Social Object Recognition

**Figure 5:**
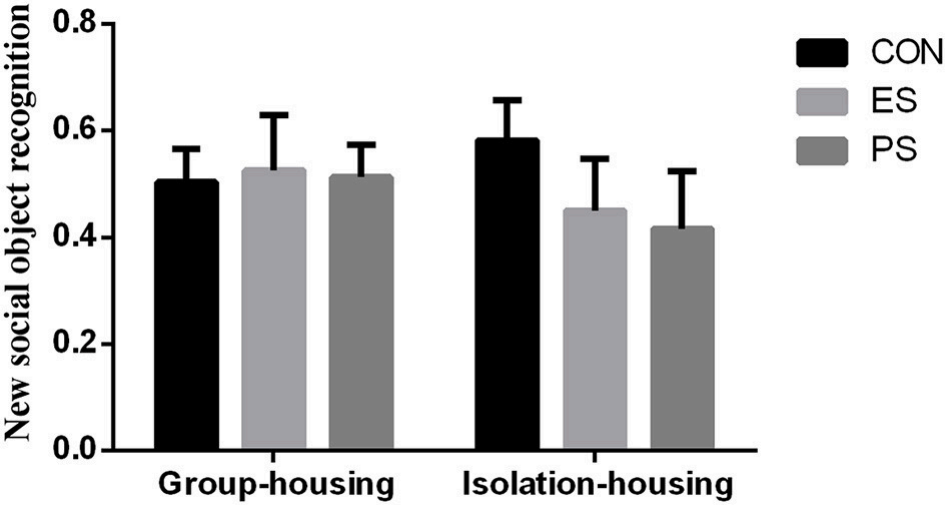
Effects of stress and housing conditions on new social object recognition. The results are expressed as the mean ± S.E.M (*n* = 7 per group). New social object recognition was calculated as (time spent in the new object zone)/(total time spent in the interaction zone) × 100%.

The result of new social object recognition is shown in Figure 5. There were no main effects of stress (*F*_(2, 37)_ = 0.28, *p* = 0.76) or housing conditions (*F*_(1, 37)_ = 0.06, *p* = 0.80), nor of stress × housing condition interaction (*F*_(2, 37)_ = 0.47, *p* = 0.63), indicating that neither stress nor the housing condition influenced the new social object recognition in our experiment.

### Effects of Stress and Housing Conditions on Social Avoidance

**Figure 6:**
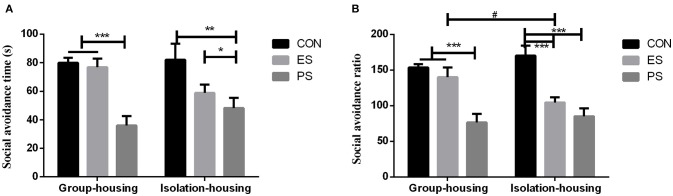
Effects of stress and housing conditions on the social avoidance time **(A)** and ratio **(B)**. The results are expressed as the mean ± S.E.M (*n* = 7 per group). The social avoidance time is calculated as time in the interaction zone with CD-1. The social avoidance ratio is calculated as (time in the interaction zone with CD-1 mice)/(time in the interaction zone without CD-1 mice) × 100%. ***p < 0.001, **p < 0.01, *p < 0.05, the differences among CON, PS and ES mice. ^#^*p* < 0.05, the difference between housing conditions in the ES group.

As shown in Figure 6, there were significant main effects of stress (time: *F*_(2, 35)_ = 16.26, *p* < 0.001; ratio: *F*_(2, 35)_ = 27.48, *p* < 0.001) and stress × housing condition interaction on the social avoidance ratio (*F*_(2, 35)_ = 3.34, *p* < 0.05). However, effects of the housing conditions (time: *F*_(1, 35) =_0.05, *p* = 0.83; ratio: *F*_(1, 35)_ = 0.14, *p* = 0.71) and stress × housing condition interaction on social avoidance time (*F*_(2, 35)_ = 2.54, *p* = 0.091) were not significant. The *post hoc* analysis revealed that both ES and PS mice exhibited a lower social avoidance ratio (Figure 6B) compared to the controls under isolation housing conditions (*p* < 0.05). However, only PS mice showed significant social avoidance under the group housing conditions (*p* < 0.05).

## Discussion

In the presents study, we aimed to develop an improved social stress model to better distinguish the effects of PS and ES. Reduced weight gain in mice exposed to PS or ES compared to the control mice, suggested that both stressors indeed induced a substantial stress response in adolescent mice. We also found that PS and ES exerted differential effects on social behaviors. Finally, we found that social support had different effects on the stress induced changes in social behavior. These results suggest that our paradigm is indeed an effective adolescent stress model that mimics the complex effects of social environmental factors on adolescent development.

Social avoidance primarily reflects a state of anxiety or fear in the context of defeat and this behavioral phenotype bears relevance for posttraumatic stress disorders, such as social phobia (19, 34). Our present study demonstrated that both PS and ES significantly increased social avoidance to previously stressful context, compared to the corresponding controls under the isolation housing condition; while only the PS, but not ES mice showed a lower social avoidance ratio, compared to that of ES mice under group housing conditions. As mentioned above, mice exposed to a social defeat in previous studies suffered compound physical and emotional social stress (19, 20). We found that “pure” physical stress and emotional social defeat can also induce contextual social avoidance, a behavioral phenotype relevant to posttraumatic stress disorder through experiencing or witnessing a traumatic event. Additionally, a more significantly decreased social avoidance ratio was observed in PS-exposed mice, indicating that physical stress had greater effects than those of emotional stress.

In contrast to social avoidance, social interaction reflects a more general social interest (35). We found that only PS exposed mice showed lower social interaction behaviors with peers, including lower interaction time and ratio with other mice, suggesting that physical social stress induced a more generalized impairment in their social behavior. Previous studies reported that physical stress induced a more extensive and robust influence on the social behavior of animals than that of emotional stress (20, 36). However, in these studies, PS was somehow conflated with ES exposure, while here we further demonstrated that pure physical social stress could impair social behavior, while only emotional social stress did not cause a general decrease in social interest. Additionally, there were no differences in the new social object recognition task between groups, suggesting that impaired social interaction could not be attributed to a reduced recognition ability. Mice in different groups also exhibited similar locomotor activity, further excluding the potential effects of less contact with peers on the evaluation of social interest in the social interaction test.

The protective effects of social support on trauma and related psychiatric disorders have been extensively reported (20, 29, 37). Partially consistent with previous studies, we also found that some of the changes induced by adolescent PS and ES were moderated by the housing conditions, isolation or group housing. For example, in the social avoidance paradigm, PS induced a lower time and ratio in the interaction area, with effects being unaffected by the housing conditions, while the social avoidance behaviors seen in ES mice were only observed in the isolation housing conditions. Similarly, the PS induced reduction in social interest was independent of the housing conditions. In other words, the protective effect of social support seems to be limited to the ES-exposed mice. Although the exact reasons for this differential effect of social support are unclear, several factors might be involved. First, only the PS group exhibited an impairment of general social interest as manifested by a decreased social interaction time and ratio. Thus, we can speculate that PS exposed mice may be less capable of effectively using social support. To verify this possibility, the daily social behaviors of group-housed PS exposed mice with cage mates, need to be investigated further in the future. Moreover, the lack of social contact is closely related to loneliness, a psychological stress that can cause a variety of behavioral and physiological changes (38, 39). Additionally, as our data showed, PS induced more severe deficits than ES did and it can be speculated that social support may not provide sufficient protection to the more severe consequences of PS. In summary, this suggested that lower availability of social support to physically stressed subjects may contribute to weakened beneficial effects in a social environment. Therefore, improving individual social support utilization is a critical issue.

Our results showed that exposure to PS or ES reduced weight gain in mice compared to the control mice, a result consistent with previous studies (40, 41). Warren et al. also found that both PS and ES reduced weight gain (20, 42, 43). Somewhat surprisingly, in PS-exposed mice, isolation housing increased body-weight gain compared to that observed in the group housing conditions. It is currently unclear why this occurred. As mentioned above, PS decreased social interactions with peers. There may be a compensatory mechanism, whereby the mice exposed to PS decreased play behavior with cage mates and increased their food intake, due to an increased basal metabolic response (43).

## Conclusion

In the present study, we developed an improved social defeat stress model that could help us further discriminate between the different effects of physical and emotional stress. We found this model to be an effective adolescent social stress model, of inducing alterations in experience-relevant social anxiety/fear and general social interaction. Importantly, these alterations were differentially affected by social support conditions after the stressful experience, depending on the type of stressor. These findings provide important evidence regarding the response to physical and emotional stressors, interacting with psychological interventions. We are confident that the model will be beneficial for understanding stress-related psychopathology.

## Author Contributions

WW designed the research; ML and HX performed the research, acquired and analyzed the data; ML, HX, and WW drafted, revised, and wrote the paper.

### Conflict of Interest Statement

The authors declare that the research was conducted in the absence of any commercial or financial relationships that could be construed as a potential conflict of interest.

## Supplementary Material

Supplementary Figure 1 Schematic representation of the box in the three-chamber social approach test.
