# Supplementary figures and images for: An Improved Model of Physical and Emotional Social Defeat: Different Effects on Social Behavior and Body Weight of Adolescent Mice by Interaction With Social Support
Source: Front Psychiatry. 2018 Dec 11;9:688. doi: 10.3389/fpsyt.2018.00688 (PMC6297843; doi:10.3389/fpsyt.2018.00688)

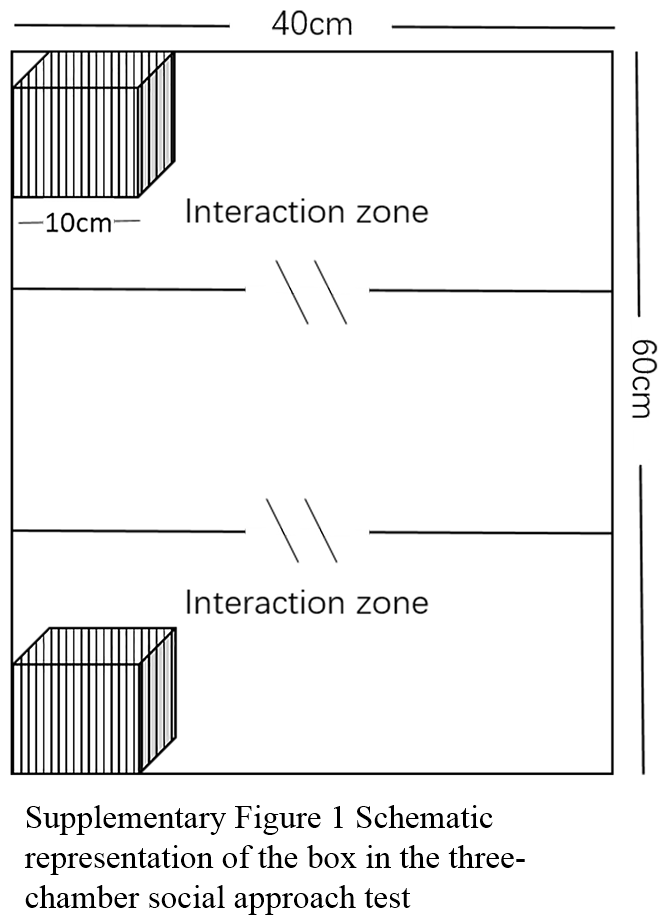

Supplement: Supplementary Figure 1 — Schematic representation of the box in the three-chamber social approach test. [file Image_1.TIF]
